# The Role of Vitamin C in Prevention of Preterm Premature Rupture of Membranes

**DOI:** 10.5812/ircmj.5138

**Published:** 2013-02-05

**Authors:** Nayereh Ghomian, Leili Hafizi, Zahra Takhti

**Affiliations:** 1 Department of Obstetrics and Gynecology, Imam Reza Hospital, Faculty of Medicine, Mashhad University of Medical Sciences, Mashhad, IR Iran

**Keywords:** Ascorbic Acid, Pregnancy, Fetal Membranes, Premature Rupture, Prevention and Control

## Abstract

**Background:**

Preterm premature rupture of membranes (PPROM) is one of the most important complications of the pregnancy and cause perinatal morbidity and mortality. History of PPROM is a risk factor of recurrent PPROM. Vitamin C plays an important role in collagen metabolism and increases resistance maintenance of the chorioamniotic membranes.

**Objectives:**

The aim of this study is to evaluate the role of vitamin C supplementation in prevention of PPROM in women with a positive history of PPROM.

**Patients and Methods:**

This clinical trial study was performed on 170 pregnant women with the history of PPROM, with singleton pregnancy and gestational age 14 weeks in Imam-Reza Hospital, Mashhad University of Medical Sciences during 2008 to 2010. They were randomly divided into two groups. The case patients received 100 mg vitamin C daily from 14th weeks of gestation. PPROM occurrence was compared between two groups as an indicator of the protective effect of vitamin C supplements.

**Results:**

PPROM occurred in 44.7% of controls and 31.8% of cases (P &lt; 0.05). PROM occurred in 34.1% of controls and 18.8% of cases (P &lt; 0.05). Pregnancy was terminated at term gestation in 21.2% of controls and 49.4% of cases (P &lt; 0.05). Rupture of membranes was significantly decreased in the case group.

**Conclusions:**

Vitamin C supplementations after 14th weeks of gestation can prevent from PPROM in women with the history of PPROM.

## 1. Background

Premature rupture of membranes (1) defined as leakage of amniotic fluid through rupturedchorioamnioticmembranes that occur before starting the labor pain at any gestational age. It is one of the most common problems in obstetrics and affects 10-20% of all pregnancies. (2,3) If PROMoccurrbefore term pregnancy (37 gestational weeks), it is named as preterm premature rupture of membranes (PPROM). Although PPROM involves 3% of pregnancies, it is the cause of one third of all preterm births with increased rates of neonatal and maternal morbidity and mortality (4). Its pathophysiologic mechanism is little known. It has been reported that premature ruptured membranes have less collagen content, which is necessary to provide mechanical strength to fetal membranes and therefore tension resistance is decreased (5). However, PPROM should be considered important because of intrauterine infection risk (6). Smoking, previous PPROM, intrauterine infection, bacterialvaginosis,multifetalgestations, cervical shortening,hydramniosis, and inadequate availability of some nutrients during pregnancy such as copper, zinc, magnesium, β-carotenes, vitamin E, and vitamin C have been identified as risk factors for PPROM and low birth-weight (7-9). Vitamin C is involved in collagen synthesis, collagen secretion, andcollagenolysisprocesses (10). Occurrence of PPROM has been associated with changed patterns of collagen synthesis and decreased concentration of vitamin C at 28th weeks of gestation (11). It is reported that PROM could be used as a functional test of vitamin C status during pregnancy (12). Some studies have shown that there were lower levels of ascorbic acid (vitamin C) in serum, leucocytes, and amniotic fluid of cases with PPROM as compared to the control group (13). But they have provided little information about the relationship between vitamin C intake and its role in PPROM. The aim of this study is to evaluate the role of vitamin C in prevention of PPROM.

## 2. Objectives

The aim of this study is to evaluate the role of vitamin C supplementation in prevention of PPROM in women with a positive history of PPROM.

## 3. Patients and Methods

One hundred and seventy pregnant women with 14 weeks gestational age and the history of at least one PPROM with singleton pregnancy were enrolled in this clinical trial, after being completely explained about study conditions and signing informed consent. The study was approved by the Ethics Committee of Mashhad University of Medical sciences, and was performed in Imam-Reza University Hospital during a time period of 2008 to 2010. Inclusion criteria was the history of at least one PPROM in previous pregnancies, body mass index (BMI) of 18.5-30 kg/m2, singleton pregnancy, normal fetus and normal amniotic fluid insonography, mother age of 18-35yrs, normal cervix length (more than 25mm), no Tobacco usage, and no consumption of vitamin C supplements. Patients were randomly divided into two groups:

For 85 women in the case group, 400µg folic acid daily was prescribed in the first trimester, then iron tablet containing 30 mg elemental iron and chewing tablet of 100 mg vitamin C (Iran-Darufactory) daily were added from 14th weeks of gestation (2) and was continued up to 37th weeks.

In control group,85patientsweretreated similar except for chewing tablet of placebo insist of same shape of vitamin C tablets. A questionnaire was completed for each woman including age, weight, history of previous disease, history of previous pregnancies, and gestational age of PPROM at previous pregnancy. The patients were evaluated for bacterialvaginosisin the first visit, moreover anytime during the survey whenever the patient has been complaining ofvaginosissigns, and if discovered it was treated medially.Sonographywas performed for all of the cases during 12-14 weeks to evaluate the length of cervix and number of fetuses. All of thepatients were evaluated monthly during the second trimester. At the end of second trimester, they were evaluated withsonographyfor the volume of amniotic fluid and fetal anomalies. The fetal membrane rupture was obtained by sterile speculum and observing amniotic fluid existing from the cervix, fern test orNitrazintest. After delivery, the questionnaire was completed by data such as the date and cause of referring to the hospital, date and time of rupture of membranes, gestational age, neonate’s sex, birth-weight, and five minute Apgar score. Data was analyzed by SPSS software (version 11). For quantitative variables, T test, and variance analysis were used if the variables were parametric inKolmograv-Smironovtest. Otherwise, Mann-Whitney and Cross-Calvaistests were used. Chi-square test was used for qualitative variables. P ≤ 0.05 wasconsidered statistically significant.

## 4. Results

There was no significant difference between case and control groups in basic interventional factors (Table 1). Rupture of membranes was significantly decreased in the case group in the shape of PPROM, PROM and during term labor. Mean latency period was significantly (P = 0.002) higher in case group (19.02 ± 6.1 hours) vs. control group (13.42 ± 6.3 hours). Both groups were statistically different in the view of gestational age, birth-weight, and neonatal Apgar score (Table 2). In the case group, 61 patients (71.8%) had vaginal delivery and 24 patients (28.2%) underwent cesarean section. it was similar in the control group, (60 women (70.6%) underwent vaginal delivery and 25 women (29.4%) cesarean section). (P = 1.007). In the next step we compared neonatal outcomes between case and control groups in specific patient who were involved PPROM or PROM. The mean birth-weight of women with PROM was significantly higher in the case group (3068 gram) compared to the control (2968 gram) (P = 0.002). The mean neonatal Apgar score in patients with PROM was 9.0 in the case group and 8.3in the control group (P = 0.03). The mean gestational age at delivery was 38.1 weeks in case group and 37.7 weeks in control group (P = 0.02). Mean of latency period was 3.6 ± 2.9 hours in case group and 3.3 ± 3.6 hours in control group (P ≤ 0.001). The mean birth-weight of women with PPROM was 2370 grams in case and 2102 grams in control group (P ≤ 0.001). The mean neonatal Apgar score was 8 in patients with PPROM in case group and 7.3 in control group (P ≤ 0.001). The mean of latency period was 59.2 ± 38.1 hours in case group and 27.5 ± 14.6 hours in control group. (P ≤ 0.001) The mean gestational age at the time of delivery was 34.7 ± 1.2 weeks in case group and 33.3 ± 1.4 weeks in control group (P ≤ 0.001). All the mentioned differences were statistically significant.

**Table 1.** Comparison of Interventional Factors Between Case and Control Groups Interventional

Interventional Factors, Mean	Case Group	Control Group	P value
**Age, y**	29.8	29	0.132
**Number of Pregnancy**	2.5	2.6	0.99
**BMI, Kg/ m^2^**	21.4	20.8	0.4
**Gestational age at PPROM in previous pregnancy**	31.05	31.4	1.70
**Number of Dead neonates**	0.3	0.6	1.88
**Number of Abortion**	0.14	0.05	0.62

**Table 2.** Comparison of Pregnancy and Neonatal OutcomesBetween Case and Control Groups

Outcome	Case Group	Control Group	P value
**Pregnancy, No. (%)**			
PPROM	27 (31.8)	38 (44.7)	0.000
PROM	16 (18.8)	29 (34.1)	0.000
Normal a	42 (49.4)	18 (21.2)	0.000
**Neonatal, Mean ± SD**			
Mean gestational age at delivery (week)	37.1 ± 1.9	35.9 ± 2.8	0.000
Mean birth weight, gram	2840 ± 382	2630 ± 529	0.000
Neonatal Apgar score	8.4 ± 0.7	7.83 ± 0.7	0.000

^a^Rupture of membrane during term delivery

## 5. Discussion

PPROM has been known as the main cause of preterm delivery and associated with increased rates of neonatal and maternal morbidity and mortality (14-16).Although it has different causes, collagen metabolism is considered as the main factor in premature rupture of membranes. Vitamin C usage during pregnancy can modulate the collagen metabolism and cause the strength ofamniochorionmembranes. There are many factors that affect the availability of vitamin C during pregnancy.15Simhanet al. reported decreased level of vitamin C in women with premature rupture of membranes (17). The results of this study showed that vitamin C usage incase group significantly increased the gestational age at delivery, neonatal Apgar score, birth-weight, and latency period. This finding was confirmed by the study ofBarretet al. He concluded that the administration of 100mg of vitamin C in pregnant women after 20th weeks of gestation can significantly decrease the incidence of PROM and PPROM (18). Siegaet al. showed that the rates of membranes rupture before 37 weeks is increased with decrease of vitaminC supplements, although the relation was not statistically significant (1). In addition, Hajifoghahaet al. reported that the usage of vitamin C supplements after 20th weeks of gestation prevents of PPROM (19). Vermilion et al. performed a study that one group of pregnant women with the history of PPROM received vitamin C along with ferrous sulfate. Although the incidence of iron shortage anemia was decreased, no difference was observed in term of PPROM between case and control group (20). Also, Casaneuvaet al. reported no significant difference between two groups in the view of vitamin C intake and PPROM (21). These different results from the results of the present study may be due to the lower volume sample in their study. In Borna et al. study no statistically significant difference was reported between vitamin C supplements and placebo groups in terms of sepsis incidence, but resemble to our study, neonatal Apgar score and birth-weight was different between two groups (22). Vitamin C is an essential nutrient, involved in several biochemical functions. It is an antioxidant that blocks the damaging effects of oxidative stress in vitro (23). Therefore, vitamin C can prevent premature rupture of membranes through its role as an antioxidant or in collagen synthesis and maintenance (24,25). The present study had some limitations, we aimed to study the independent effect of vitamin C, but because the serum level of vitamin C was not assayed, isolation of the effect of a single nutrient is difficult. We propose a relationship between low vitamin C intake and an increased risk of preterm premature rupture of membranes. Vitamin C supplement is recommended to beadministered for pregnant women with the history of PPROM during pregnancy to prevent PPROM. Nevertheless, further studies with larger sample size and fewer limitations are needed to best clarify the role of vitamin C in prevention of PPROM especially in women with other risk factors of PPROM.
